# Evidence on the association of overall dietary factors, selected environmental, medical, demographic, and biological factors and developmental defects of enamel, including MIH and enamel fluorosis

**DOI:** 10.3389/froh.2025.1616109

**Published:** 2025-12-11

**Authors:** L. Al Dehailan, E. A. Martinez-Mier

**Affiliations:** 1 Department of Restorative Dental Sciences, College of Dentistry, Imam Abdulrahman Bin Faisal University, Dammam, Saudi Arabia; 2 Department of Dental Public Health and Dental Informatics, Indiana University School of Dentistry, Indiana University, Indianapolis, IN, United States

**Keywords:** fluorosis, molar incisor hypomineralisation (MIH), developmental enamel defects, scoping review, demographic factors, DDE, nutrition

## Abstract

**Introduction:**

Developmental defects of enamel (DDE) encompass a spectrum of conditions that occur during tooth formation, including enamel fluorosis, molar–incisor hypomineralization (MIH), and other forms of enamel hypoplasia. It has been proposed that DDE are associated with nutritional deficiencies as well as environmental exposures during tooth development.

**Objective:**

This scoping review summarized and analyzed the evidence on the association between dietary habits, environmental exposures, medical/health-related factors, demographic factors and biological factors and DDE aiming to provide a comprehensive overview of the available evidence in this area.

**Methodology:**

Following the Joanna Briggs Institute (JBI) framework using Population, Concept, and Context (PCC) to guide the development of the research question and eligibility. The population of interest were individuals from any age group or gender diagnosed with DDE. The eligibility concepts were factors that may contribute to DDE such as dietary and environmental exposures. The study selection followed the PRISMA guidelines. Studies published from January 1993 to December 2024 were identified through searches in Web of Science and PubMed.

**Results:**

Our review included 125 studies from 1993 to 2024, mainly on fluorosis (105 studies), mostly cross-sectional, and conducted in Asia and North America. Fewer studies addressed MIH (5) and other non-fluorosis DDE (15), primarily in Europe, South America, and Asia, with most participants being children aged 6–12 years, and small sample sizes. The review evaluated DDE and its main subtypes, molar-incisor hypomineralization (MIH) and enamel fluorosis across conditions, overlapping risk factors were identified, such as excessive fluoride intake, vitamin D deficiency, early childhood illnesses, and exposure to environmental contaminants. Condition-specific patterns were also noted, fluorosis being primarily associated with high fluoride exposure and early weaning, whereas MIH was more frequently linked to vitamin D deficiency and early systemic

**Conclusion:**

The findings highlight that enamel fluorosis, MIH, and other enamel hypoplasias are part of a shared continuum of DDE influenced by interrelated dietary, environmental, and biological factors. These findings suggest common developmental pathways leading to enamel disruption and emphasize the need for longitudinal and mechanistic studies to clarify causal relationships and inform preventive strategies.

## 1 Introduction

Developmental defects of enamel (DDE) represent an overarching category of enamel disturbances that occur during tooth development. Within this spectrum are specific subtypes, including enamel fluorosis and molar–incisor hypomineralization (MIH), as well as other forms such as enamel hypoplasia, amelogenesis imperfecta, and enamel defects associated with systemic or local factors. Although these defects share similar developmental origins, they differ in etiology and clinical presentation. These defects can result in abnormalities such as thin enamel, discolored spots, and pitting or grooves on the enamel surface. They can be caused by a combination of genetic, environmental, systemic, and local factors. Systemic factors associated with DDE include nutritional deficiencies, metabolic disorders, systemic infections, and exposure to medications or environmental toxins. Local factors include trauma and injury to the developing tooth that disrupts the normal formation of enamel (1, 2). Because fluorosis and MIH are the most frequently investigated forms of DDE in the literature, this review focuses primarily on these two conditions while also considering evidence related to other DDE where available to provide a comprehensive overview of the field.

Nutritional deficiencies, particularly low intake of calcium, vitamins, as well as excessive fluoride consumption during tooth development, can increase the likelihood of DDE. Inadequate maternal nutrition during pregnancy and insufficient nutrition in early childhood have also been associated with a higher risk of DDE. Furthermore, exposure to environmental toxins, such as elevated levels of heavy metals in the diet, may contribute to the development of DDE (1, 2).

MIH is a specific type of DDE that primarily affects the first permanent molars and incisors. The exact cause of MIH is still unknown, but various dietary factors, including early childhood feeding practices, have been suggested to play a role in its development. Certain studies have found an association between prolonged breastfeeding and the occurrence of MIH. Additionally, it has been suggested that low levels of vitamin D may been linked to an increased risk of MIH, however, further investigation is needed to prove such causation (3, 4).

Among the subtypes of DDE, enamel fluorosis is one of the most extensively studied. It affects the appearance and structure of dental enamel. Unlike other DDE, the etiology of enamel fluorosis has been extensively studied and is primarily attributed to the excessive consumption of fluoride during tooth development. Excessive intake of fluoride has not only been linked to an increased risk of developing enamel fluorosis but also to other developmental enamel defects such as enamel hypoplasia and MIH (5–8). Multiple studies have indicated that children may be consuming excessive amounts of fluoride through dietary sources like infant formula and certain foods and soft drinks (9, 10). Accordingly, in this review, DDE is treated as a comprehensive construct encompassing enamel fluorosis, MIH, and other developmental enamel disturbances.

While some etiological factors for DDE have been studied in more detail and their mechanisms are better understood, others are still being investigated. Certain nutritional deficiencies, such as inadequate intake of calcium, and vitamin D have been proven to affect the mineralization and formation of tooth enamel. However, the exact mechanisms by which these deficiencies cause DDE are complex and involve interactions between biological processes and nutrient availability (4).

Similarly, poor maternal nutrition during pregnancy and inadequate nutrition during early childhood have been suggested as potential causes of DDE. Nutritional deficiencies during critical periods of tooth development may impact enamel formation and result in enamel defects. However, the specific mechanisms linking poor maternal nutrition and inadequate childhood nutrition to DDE are also still being investigated (11).

The role of environmental toxicants, including high levels of heavy metals in the diet, in the development of DDE has also been studied (12). Heavy metals can disrupt enamel formation and affect the quality of tooth enamel. However, the precise mechanisms by which these environmental toxicants contribute to DDE are not fully understood and require further research.

While multiple associations between dietary factors and the development of DDE have been established, establishing definitive causality will require extensive research and the consideration of various confounding factors. Additionally, the interaction and potential synergistic effect of dietary factors and genetic and environmental influences needs to be better understood.

Given the fact that these factors and their association to DDE have been studied at varying degrees of detail, it seemed pertinent to identify what evidence is available in this area. We conducted a scoping review of available literature to summarize and evaluate the evidence on the association between dietary habits, environmental exposures, medical/health-related factors, demographic factors, and biological factors, and DDE, with a special emphasis on fluorosis and MIH. The study design, outcome measures, analytical methods, and location of the included studies were examined. This scoping review aimed to provide a comprehensive overview of the available evidence in this area.

## 2 Materials and methods

Scoping reviews (also known as mapping reviews) are exploratory research projects that systematically map the literature on a topic by identifying key concepts, theories and sources of evidence that inform practice.

This scoping review was conducted using the Joanna Briggs Institute (JBI) methodology and guided by the Population, Concept, and Context (PCC) framework. The population of interest included individuals of any age or sex with clinically diagnosed DDE, including enamel fluorosis, MIH, and other enamel hypoplasias. The review aimed to map the range of dietary, environmental, medical, demographic, and biological factors associated with DDE as a whole, while placing particular emphasis on fluorosis and MIH due to their predominance in the published evidence (13). The population of interest included individuals of any age or sex with clinically diagnosed DDE, including enamel fluorosis, MIH, and other enamel hypoplasias. The review aimed to map the range of dietary, environmental, medical, demographic, and biological factors associated with DDE as a whole, while placing particular emphasis on fluorosis and MIH due to their predominance in the published evidence.

Research Question:

To explore the factors associated with DDE in the population.

Eligibility Criteria:

Population: Studies conducted on individuals of any age group or gender who have been diagnosed with DDE.

Concept: Various factors contribute to the development of DDE. This includes factors such as dietary habits, environmental exposures, medical/health-related factors (e.g., use of supplements, medications), demographic factors (e.g., socioeconomic status, age, gender), and biological factors (e.g., prematurity, birth weight, nutritional status).

Context: Studies conducted in any geographic location or setting, including both clinical and community-based settings.

By using the Population, Concept, and Context (PCC) framework, this scoping review aimed to gather a comprehensive understanding of the factors associated with dental fluorosis, dental enamel hypoplasia, and MIH across different populations, concepts, and contexts.

### 2.1 Eligibility criteria

Based on the JBI approach, the inclusion and exclusion criteria were developed to help selecting appropriate papers (14).

**Figure 1:**
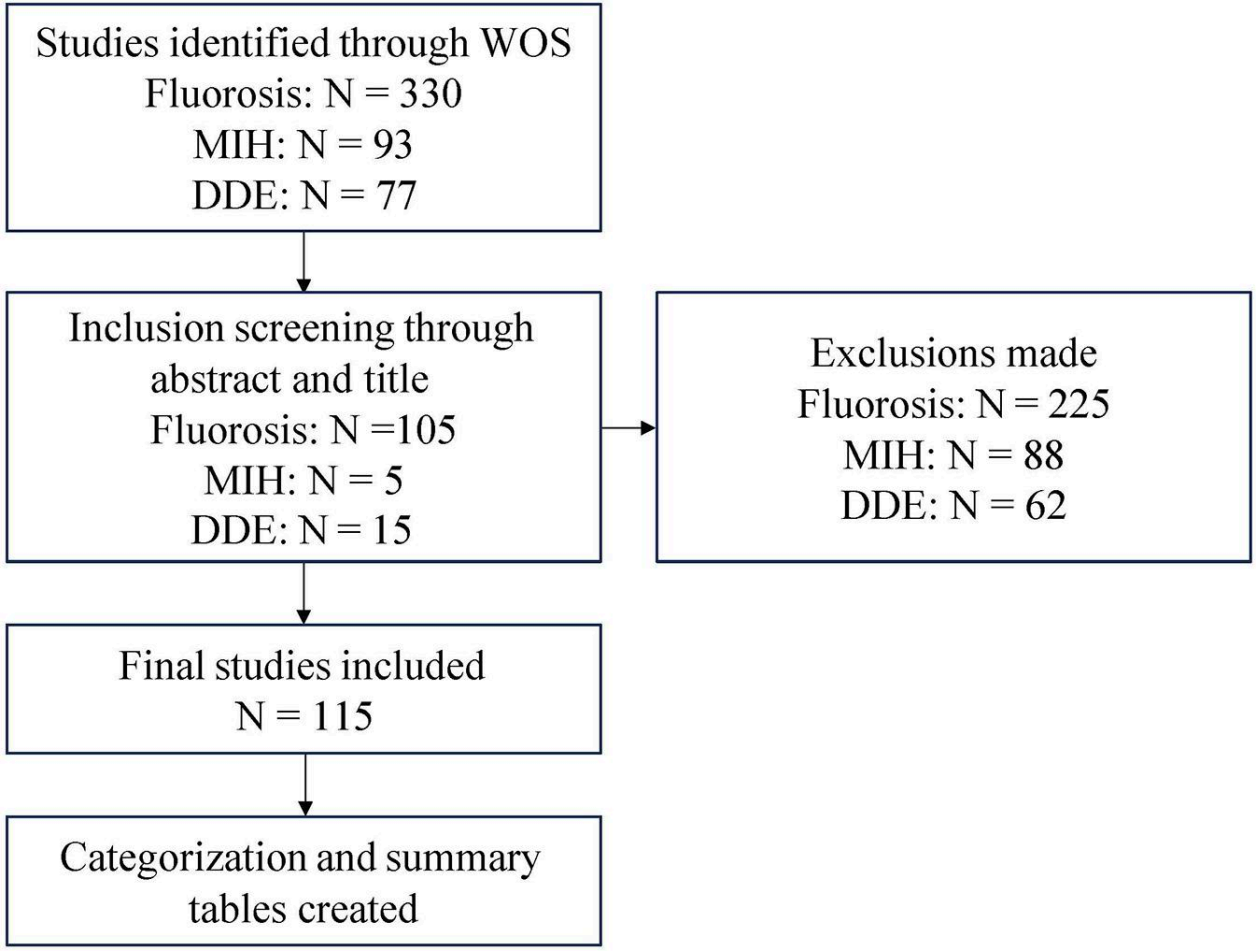
Flowchart of the study screening and selection process.

The inclusion criteria for our review were as follows: (1) published or recently accepted full-text articles from January 1993 until December 2024 (2) articles investigating the association between data-driven dietary factors and enamel defects outcomes, including fluorosis, MIH and non-fluoride opacities assessed via clinical examination, photographs, questionnaires, or secondary data analysis (3) participants at any age from child to elderly (4) English language articles. The exclusion criteria were as follows: (1) studies involving participants suffering from any chronic conditions or disabilities (2) animal studies (NOT ALL = (animal OR rat OR mouse OR “zebra fish” OR rabbit) & (3) review articles. Figure 1 presents a flowchart summary for the screening process used to identify eligible articles.

### 2.2 Search strategy

We utilized the checklist from PRISMA Extension for Scoping Reviews (PRISMA-ScR: Checklist and Explanation Annals of Internal Medicine).

Our scoping review process was built upon a framework first introduced by Arkey and O'Malley and later modified by Levac et al. and Calquhoun et al. (15–17). The process described is to first develop a research question, finding and identifying all relevant studies, creating summary charts, and then summarizing the findings (15). Our search was conducted on Web of Science (WOS) as provided by Indiana University with a second search conducted using Pub Med.We selected a series of terms and parameters to find studies within the scope of our review. The terms used in the search were either constant terms utilized to ensure relevancy, or a set of three overarching categories with subcategories to find relevant information.

Our search strategy focuses on locating relevant published research in the previously mentioned databases. Using a combination of key terms, and the Boolean operators “AND”, and “OR”, we defined a general strategy applicable for PubMed/Medline. The overarching categories used were Fluorosis, MIH, and (overall) DDE. We decided to conduct independent searches for fluorosis and MIH, as they are the type of DDE most studied independently. A third category of DDE was created, and it included studies that investigated more than one specific type of DDE. The subcategories used are prevalence, diet, “dietary exposure”, tetracycline, anti-bacterial agents, and infant food. The constant terms used to ensure that the search remained relevant being: TS = (Dental) AND DOP = (1900-01-01/2023-01-01) AND ALL = (“clinical examination” OR index OR score OR exam) AND LA = (English) AND ALL = (children OR pediatric OR infant) NOT ALL = (diabetes OR renal OR dialysis OR “chronic condition”) NOT ALL=(animal OR rat OR mouse OR “zebra fish” OR rabbit) NOT DT = (Review OR Database Review)). Additionally, age was considered with the terms: Infant, Newborn (0–28 do) infant (1–23 mo) Child, Preschool (2–5 yo) Child (6–12 yrs) Adolescent (13–18 yo). The studies gathered where then individually relevance sorted utilizing the abstract title and WOS keywords. Example outputs for WOS searches are as follows: “Fluorosis, Dental AND (Diet OR Dietary Exposure OR Fluorides OR Fluoridation OR Tetracycline OR Anti-Bacterial Agents OR Infant Food OR Infant Formula OR Prevalence) Limiter—Child: birth-18 years” and “Fluorosis, Dental AND (Diet OR Dietary Exposure OR Fluorides OR Fluoridation OR Infant Food OR Infant Formula OR Prevalence) AND (Infant, Newborn OR Infant OR Child, Preschool OR Child OR Adolescent)”.

### 2.3 Data charting and synthesis

After conducting the initial search, electronic copies of all eligible articles were obtained. The titles and abstracts of these articles were reviewed by both LAD and EAMM. Full texts were then read by both, and reference lists were manually searched for any additional relevant papers.

A data extraction form was then adapted from JBI to record key information relevant to the research question. A narrative summary of the included studies was categorized based on the key parameters, such as authors, country of origin, aims, study description and key findings. All the selected articles were summarized (Supplementary Table 1).

No formal review protocol was prospectively registered; this was acknowledged as a limitation to ensure methodological transparency.

## 3 Results

### 3.1 Characteristics of the studies

Our review encompassed studies published between 1993 and 2024. Our initial search through WOS and PubMed identified 500 records, of which 125 met the inclusion criteria. Among these, the majority examined enamel fluorosis (*n* = 105), predominantly cross-sectional studies (*n* = 86) conducted across diverse regions such as Asia (37 studies) and North America (23 studies). A smaller number of studies addressed molar–incisor hypomineralization (MIH; *n* = 5), mainly from Europe and South America, while 15 studies evaluated other, non-fluorosis developmental enamel defects within the DDE spectrum. Together, these studies represent the full range of developmental defects of enamel (DDE) considered in this review. Most investigations employed cross-sectional designs (*n* = 99), with participants primarily children aged 6–12 years for fluorosis and other DDE, and children or adolescents for MIH. Accordingly, the review examined DDE as an overarching category encompassing fluorosis, MIH, and other enamel hypoplasias to identify shared and condition-specific determinants. The sample sizes varied across studies, with the majority involving smaller sample sizes ranging from 1 to 100 participants.

#### 3.1.1 Outcome measures for DDE and its subtypes

##### 3.1.1.1 MIH outcome measures

For MIH, the European Academy of Pediatric Dentistry criteria (*n* = 3), Modified Developmental Defects of Enamel Index (*n* = 2), and Thylstrup and Fejerskov Index (*n* = 1) were used for diagnosis, identification, and screening. Serum 25(OH)D concentrations were also measured and used as a biomarker. Three studies used MIH as their clinical outcome and one used HSPM.

#### 3.1.2 Fluorosis outcome measures

As a result of our scoping review, we identified various outcome measures used to assess DDE, including fluorosis, molar-incisor hypomineralization (MIH). For fluorosis, clinical exams were conducted using different indices such as the Surface Index of Fluorosis (TSIF) (*n* = 5), Modified TSIF (*n* = 1), Dean's index (*n* = 16), Modified Dean's index (*n* = 2), Fluorosis Risk Index (FRI) (*n* = 3), Thylstrup-Fejerskov (TF) index (*n* = 23), Community Fluorosis Index (CFI) (*n* = 2), Modified CFI (*n* = 1), Developmental Defects of Enamel (DDE) index (*n* = 1), and Modified DDE index (*n* = 1).

The articles included in our review used surveys and questionnaires to gather information on fluoride exposure, fluoride history, fluoride ingestion, toothpaste use, dietary fluoride intake, aesthetic concerns, intelligence, milk tea consumption, and nutritional status. Additionally, images and photographic assessments were utilized to evaluate fluorosis through blind scoring, photographic examination, and assessment of fluoride content. In total, 78 studies utilized clinical exams, 27 included surveys or questionnaires and 3 utilized images and photographic assessment.

#### 3.1.3 DDE (other than MIH and fluorosis) outcome measures

In the case of DDE, the color and structural changes in teeth, the developmental defects of enamel index (*n* = 7), the modified developmental defects of enamel index (*n* = 2), and the Dean's index (*n* = 1) were used to assess clinical outcomes. In these studies, various conditions were assessed, including general “enamel effects”, enamel fluorosis, deciduous molar hypomineralization (DMH), non-fluoride related DDE, MIH, enamel opacities.

### 3.2 Factors associated to the development of DDE

#### 3.2.1 DDE factors

The articles included in our review examined various factors that may contribute to the development of defects in enamel. Our review focused on articles that investigated dietary factors, but many of them included others. We categorized these other factors as environmental, medical/health-related, demographic, and biological. Because our inclusion criteria focused on nutritional factors, our review of these other factors cannot be considered comprehensive.

In terms of dietary factors, the articles explored the impact of fluoride exposure from diet, current breastfeeding practices, not exclusively feeding breast milk, and water and milk intake. Nutritional deficiencies including inadequate intake of essential nutrients during tooth development, children who suffered from intrauterine malnutrition improper nutrition during fetal development and survivors after prenatal intra-uterine transfusions (for rhesus incompatibility for example). These nutritional deficiencies, including inadequate intake of essential vitamins, were found to be associated with an increased risk of enamel defects.

Environmental factors that were examined included water fluoridation history, fluoride concentration in drinking water (measured directly and mentioned in two studies), the presence of fluoridated water programs, and residential location. Exposure to environmental toxins, such as lead, that can disrupt enamel development and contribute to the formation of enamel defects was also studied. These factors were either measured directly or reported.

The articles also investigated medical/health-related factors such as surgical procedures and orthodontic treatments (reported), enamel hypoplasia (measured directly and mentioned in three studies), the use of antibiotics during pregnancy (reported), the use of penicillin and cephalosporins in early childhood (reported), early illness episodes (reported), asthma/bronchitis in the first four years of life (reported), neonatal health conditions (reported), jaundice at birth (reported), frequent vomiting episodes (reported), infant/childhood disease (reported), systemic illness during the first 5 years of life (reported), trauma or infection on deciduous teeth (reported), prolonged use of fluoride tablets (reported), and hospitalization in the first year of life (reported).

Demographic factors that were considered included maternal age participants' age (mentioned in two studies), gender, all measured directly, socio-economic status (indexed measure), and ethnicity (reported).

Lastly, the articles explored biological factors such as premature birth/low birth weight (measured directly), birth weight (measured directly), prematurity (reported), maternal factors during pregnancy (reported), and nutritional status (reported), including malnutrition.

It is important to note that fluoride exposure can be classified as both a dietary and an environmental factor. Fluoride may be consumed through food, beverages, and dental products (dietary exposure), and it may also be present in the community water supply or environment based on regional fluoridation practices (environmental exposure).

**Figure 2:**
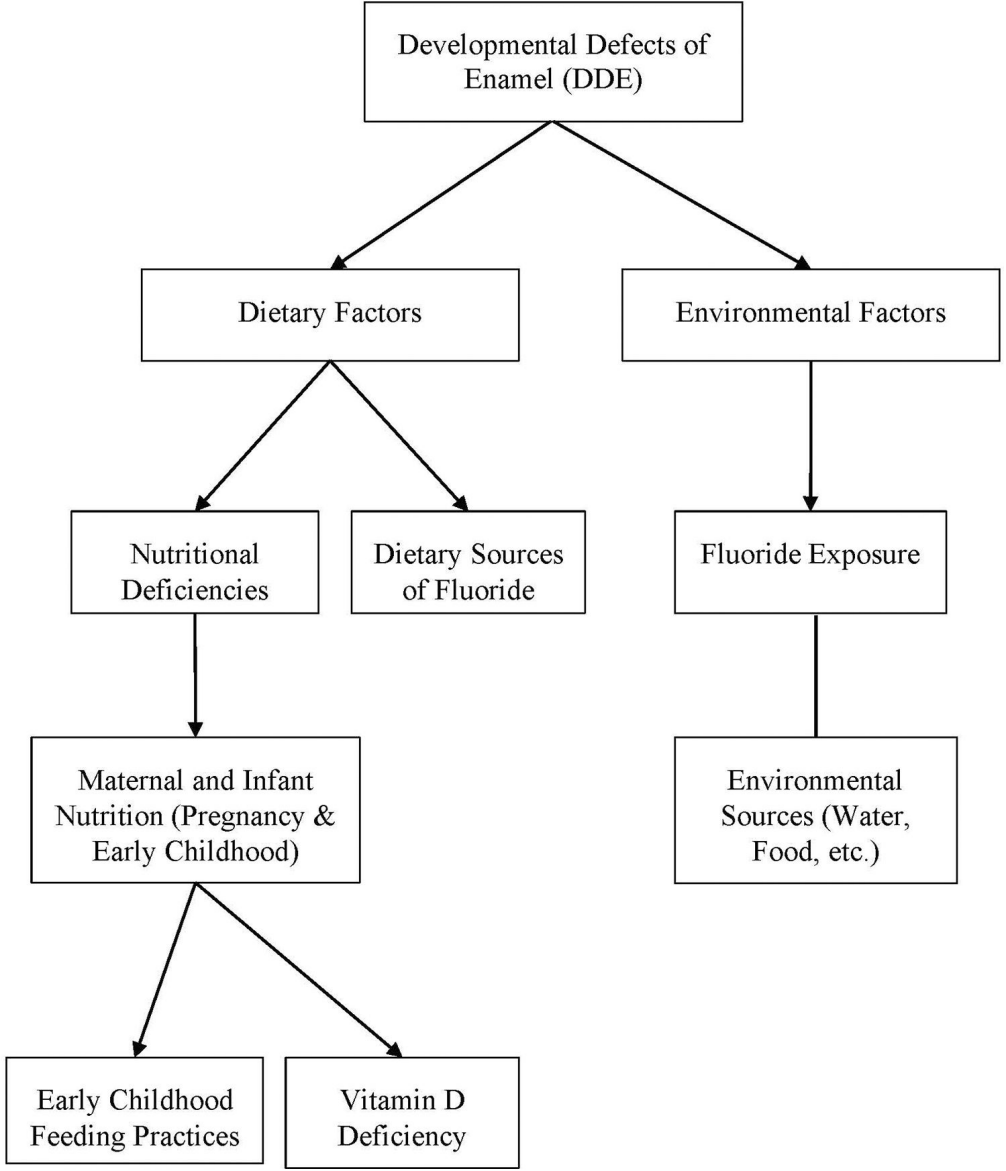
Summary of factors associated with DDE.

A consistent set of risk factors emerged as relevant to the development of all the conditions studied in the review. While each condition has specific distinctions, there is substantial overlap in the contributing factors, suggesting shared pathways in enamel disruption during tooth development. A summary of factors associated with DDE can be found in Figure 2.

#### 3.2.2 Common factors across all DDE conditions

Dietary factors most frequently reported included excess fluoride intake from diet (e.g., high fluoride drinking water, formula, or food products), inadequate intake of essential nutrients (particularly vitamin D, calcium, and phosphorus), and suboptimal early feeding practices such as non-exclusive breastfeeding. These factors were measured through both direct assessment and self-reported data.

Environmental exposures, notably fluoride concentration in community water supplies, were central to the development of fluorosis and also reported in MIH and general DDE studies. Fluoridation history and geographic residence were often used as proxies.

Medical and health-related factors reported across conditions included early childhood illnesses, systemic infections, neonatal conditions (e.g., jaundice), and frequent use of antibiotics, particularly penicillin and cephalosporins. Some studies also explored associations with surgical interventions and orthodontic treatment history.

Demographic variables, such as maternal age at childbirth, child's age and gender, socioeconomic status, and ethnicity, were assessed as contextual influences. These often mediated exposure to dietary and environmental risk factors.

Biological indicators, including birth weight, prematurity, breastfeeding history, and overall nutritional status, were commonly examined. Vitamin D levels during pregnancy and early life were frequently investigated in MIH studies.

#### 3.2.3 Condition-specific observations

While most studies pointed to common risk domains, a few condition-specific patterns emerged:

Dental fluorosis was most strongly associated with high fluoride intake during early childhood, particularly through drinking water and toothpaste ingestion. Longer residence in high-fluoride regions and early weaning were also reported.

MIH studies emphasized the role of vitamin D deficiency and systemic illnesses during the first years of life. Associations with antibiotic use, particularly cephalosporins, were more frequently cited here than in other DDE studies. MIH was also examined in relation to neurobehavioral symptoms (e.g., hyperactivity), parental medication use, and maternal education level.

General DDE studies often combined multiple enamel outcomes, with less clarity on etiology. They highlighted multi-factorial influences, including nutritional deficiencies, intrauterine growth issues, and exposure to environmental toxins like lead.

A side-by-side comparison of shared and unique risk factors across these conditions is provided in Table 1.

**Table 1:** Summary of risk factors associated with DDE including fluorosis and MIH.

Risk factor	Reported in DDE	Reported in fluorosis	Reported in MIH	Strength of association of risk factor
Excess fluoride intake	Yes	Yes	Yes	Strongest evidence in fluorosis; also noted in DDE and MIH studies
Water fluoridation levels	Yes	Yes	Yes	Strongest evidence in fluorosis; also noted in DDE and MIH studies
Nutritional deficiencies (e.g., Vitamin D, Calcium, Phosphorus)	Yes	Yes	Yes	Strongest evidence in fluorosis; also noted in DDE and MIH studies
Breastfeeding practices	Yes	Yes	Yes	Strongest evidence in fluorosis; also noted in DDE and MIH studies
Use of antibiotics (e.g., penicillin, cephalosporins)	Yes	Yes	Yes	Strongest evidence in fluorosis; also noted in DDE and MIH studies
Early childhood illness/systemic conditions	Yes	Yes	Yes	Strongest evidence in fluorosis; also noted in DDE and MIH studies
Neonatal conditions (e.g., jaundice, low birth weight)	Yes	Yes	Yes	Strongest evidence in fluorosis; also noted in DDE and MIH studies
Socioeconomic status	Yes	Yes	Yes	Strongest evidence in fluorosis; also noted in DDE and MIH studies
Maternal factors (e.g., age, nutrition)	Yes	Yes	Yes	Strongest evidence in fluorosis; also noted in DDE and MIH studies
Environmental toxins (e.g., lead)	Yes	Yes	No	Strongest evidence in fluorosis; also noted in DDE and MIH studies
Neurodevelopmental symptoms (e.g., hyperactivity)	No	No	Yes	Strongest evidence in fluorosis; also noted in DDE and MIH studies
Parental medication use	No	No	Yes	Strongest evidence in fluorosis; also noted in DDE and MIH studies
Maternal education level	No	No	Yes	Strongest evidence in fluorosis; also noted in DDE and MIH studies

## 4 Discussion

Taken together, the results of our review show that the available evidence indicates that dietary environmental, and biological factors intersect in shaping the risk of DDE. These findings are consistent with prior reviews showing that disturbances in mineral metabolism, fluoride exposure, and systemic health during the secretory and maturation stages of amelogenesis contribute to enamel abnormalities (7, 18). Across the DDE spectrum, nutritional influences, particularly vitamin D, calcium, and protein intake have been highlighted as critical modulators of ameloblast function (2, 3). Our results reinforce these mechanisms by showing overlapping risk factors for fluorosis, MIH, and other DDE, suggesting that similar biological pathways may underlie distinct clinical manifestations. Overall, dietary factors such as fluoride exposure, breastfeeding practices, and water and milk intake have been associated with the development of DDE. Additionally, previous surgical procedures and the use of antibiotics during pregnancy have also been identified as contributing factors common to all DDE.

Within the DDE spectrum, enamel fluorosis remains the most extensively studied DDE subtype. Our synthesis supports previous epidemiologic and mechanistic findings that excessive fluoride exposure from water, diet, or early-life formula feeding is the dominant determinant of fluorosis severity (18, 19). For MIH, emerging evidence points to nutritional and systemic factors—especially vitamin D deficiency, childhood illness, and perinatal complications—as key contributors (4, 8, 19). The co-occurrence of these factors in many of the included studies underscores how dietary and environmental domains are intertwined rather than mutually exclusive.

Specifically, for dental fluorosis, factors such as fluoride concentration in drinking water, use of fluoride supplements, and duration of residence in a place with high water fluoride content were found to be associated with its development. Socioeconomic status, family income, obesity, age of weaning, and exposure from birth to 4 years were also identified as demographic factors related to dental fluorosis. It is also very evident that fluorosis is the most studied of DDE. This is due to the public health significance of ensuring optimal levels of fluoride intake for caries prevention.

For MIH, certain dietary habits were identified as potential contributors. Environmental factors such as fluoridation levels of water in the residential area and fluoride levels in drinking water were also found to be associated with MIH. Medical/health-related factors such as hyperactivity/inattention symptoms and frequent use of certain antibiotics were also identified as potential factors.

Vitamin status, specifically vitamin D deficiencies, were investigated for their role in DDE development, specifically MIH. While some studies suggested a link, the evidence remains inconclusive. This disagreement is likely due to lack of standardized research methodologies, differences in vitamin D assessment methods, and the complex multifactorial etiology of DDE. Proving direct causality would require a large number of longitudinal studies evaluating maternal vitamin D and following up their offsprings.

This review revealed several key findings. Firstly, studies were conducted primarily in Asia and North America, Given the fact that environmental factors play a role, location of study seems relevant. There is also no agreement on the ideal age to study these conditions, with participants ranging from children aged 6–12 years to adolescents. Study designs could be improved, most studies have been conducted with small sample sizes, were predominantly cross-sectional and did not utilize complex statistical methods to assess complex mixtures of exposures.

In conclusion, dietary factors were found to play a significant role in the development of these conditions. Fluoride exposure from the diet, consumption of certain foods and beverages, and breastfeeding practices were identified as contributing factors. Environmental factors, such as fluoride concentration in drinking water and water fluoridation history, were reported to influence the development of dental fluorosis and MIH. Medical/health-related factors, including the use of fluoride supplements, surgical procedures, and certain medications, were also identified as contributors. Demographic factors, such as socioeconomic status, age, and gender, may also play a role. Additionally, biological factors, including prematurity, birth weight, and nutritional status, can influence the development of these conditions.

Although our review shows consistent patterns (i.e., fluoride, vitamin D, illness) across DDE types, longitudinal and mechanistic evidence remains limited. Most available studies are cross-sectional, restricting causal inference and the ability to differentiate between transient developmental disturbances and chronic exposures. Prospective cohort studies integrating dietary biomarkers, environmental monitoring, and genetic susceptibility markers would help clarify dose–response relationships for fluoride, vitamin D, and trace elements. Standardized diagnostic criteria and unified reporting of DDE subtypes are also needed to improve comparability across populations.

Overall, this review highlights that enamel fluorosis, MIH, and other DDE are interconnected outcomes within a continuum of enamel development disturbances. Dietary and environmental exposures appear to operate through shared biological pathways, altered calcium and phosphate homeostasis, oxidative stress, and impaired protein removal during enamel maturation. Recognizing these overlapping mechanisms provides a foundation for preventive strategies targeting maternal and early-childhood nutrition, as well as safe fluoride exposure. Our review suggests that while multiple factors have been studied, further research is needed to better understand the potential interactions and synergies between these factors.

## 6 Limitations

The search was restricted to online sources, and only English-written papers were included in the study, which may have excluded papers from non-English-speaking nations. Additionally, the scoping review is a type of literature review that aims to map out the existing research on a particular topic. In accordance with JBI Scoping Review guidance, it is not mandatory to assess the quality of the evidence, which means that the findings may be influenced by the quality of the studies reviewed.

Another limitation of the studies reviewed is that most are small, cross-sectional studies with varying methodologies, limiting causal inference. A further limitation is that no formal review protocol was prospectively registered, which may limit reproducibility; however, this was transparently acknowledged, and the review process was conducted following JBI and PRISMA-ScR guidance to maintain methodological rigor.

## 7 Conclusion

This scoping review highlights the significant role of dietary and environmental factors in the development of developmental defects of enamel (DDE), encompassing enamel fluorosis, molar–incisor hypomineralization (MIH), and other related enamel anomalies. By recognizing these conditions as interrelated manifestations within the DDE spectrum, future research can better identify shared mechanisms and preventive strategies. Fluoride intake from food, beverages, water, and early feeding practices emerged as the most studied and consistently associated factor, particularly for fluorosis. Nutritional deficiencies, such as inadequate levels of vitamin D and calcium, along with environmental exposures and certain medical or biological conditions, also contribute to the risk of DDE. Despite these associations, much of the existing research relies on small, cross-sectional studies with varying methodologies, limiting causal inference. Further longitudinal and mechanistic studies are needed to clarify how these risk factors interact and to inform preventive strategies that promote optimal enamel development.

## Data Availability

The original contributions presented in the study are included in the article/Supplementary Material, further inquiries can be directed to the corresponding author.
